# Correlation between Alzheimer’s disease and type 2 diabetes using non-negative matrix factorization

**DOI:** 10.1038/s41598-021-94048-0

**Published:** 2021-07-27

**Authors:** Yeonwoo Chung, Hyunju Lee, the Alzheimer’s Disease Neuroimaging Initiative, Michael W. Weiner, Paul Aisen, Ronald Petersen, Cliford R. Jack, William Jagust, John Q. Trojanowki, Arthur W. Toga, Laurel Beckett, RobertC. Green, Andrew J. Saykin, John Morris, Leslie M. Shaw, Zaven Khachaturian, Greg Sorensen, Maria Carrillo, Lew Kuller, Marc Raichle, Steven Paul, Peter Davies, Howard Fillit, Franz Hefti, Davie Holtzman, M. Marcel Mesulam, William Potter, Peter Snyder, Tom Montine, Ronald G. Thomas, Michael Donohue, Sarah Walter, Tamie Sather, Gus Jiminez, Archana B. Balasubramanian, Jennifer Mason, Iris Sim, Danielle Harvey, Matthew Bernstein, Nick Fox, Paul Thompson, Norbert Schuf, Charles DeCArli, Bret Borowski, Jef Gunter, Matt Senjem, Prashanthi Vemuri, David Jones, Kejal Kantarci, Chad Ward, Robert A. Koeppe, Norm Foster, Eric M. Reiman, Kewei Chen, Chet Mathis, Susan Landau, Nigel J. Cairns, Erin Householder, Lisa Taylor-Reinwald, Virginia Lee, Magdalena Korecka, Michal Figurski, Karen Crawford, Scott Neu, Tatiana M. Foroud, Steven Potkin, Li Shen, Kelley Faber, Sungeun Kim, Lean Tha, Richard Frank, John Hsiao, Jefrey Kaye, Joseph Quinn, Lisa Silbert, Betty Lind, Raina Carter, Sara Dolen, Beau Ances, Maria Carroll, Mary L. Creech, Erin Franklin, Mark A. Mintun, Stacy Schneider, Angela Oliver, Lon S. Schneider, Sonia Pawluczyk, Mauricio Beccera, Liberty Teodoro, Bryan M. Spann, James Brewer, Helen Vanderswag, Adam Fleisher, Daniel Marson, Randall Grifth, David Clark, David Geldmacher, John Brockington, Erik Roberson, Marissa Natelson Love, Judith L. Heidebrink, Joanne L. Lord, Sara S. Mason, Colleen S. Albers, David Knopman, Kris Johnson, Hillel Grossman, Efe Mitsis, Raj C. Shah, Leyla deToledo-Morrell, Rachelle S. Doody, Javier Villanueva-Meyer, Munir Chowdhury, Susan Rountree, Mimi Dang, Ranjan Duara, Daniel Varon, Maria T. Greig, Peggy Roberts, Yaakov Stern, Lawrence S. Honig, Karen L. Bell, Marilyn Albert, Chiadi Onyike, Daniel D’Agostino, Stephanie Kielb, James E. Galvin, Brittany Cerbone, Christina A. Michel, Dana M. Pogorelec, Henry Rusinek, Mony J. de Leon, Lidia Glodzik, Susan De Santi, Kyle Womack, Dana Mathews, Mary Quiceno, P. Murali Doraiswamy, Jefrey R. Petrella, Salvador Borges-Neto, Terence Z. Wong, Edward Coleman, Allan I. Levey, James J. Lah, Janet S. Cella, Jefrey M. Burns, Russell H. Swerdlow, William M. Brooks, Steven E. Arnold, Jason H. Karlawish, David Wolk, Christopher M. Clark, Liana Apostolova, Kathleen Tingus, Ellen Woo, Daniel H. S. Silverman, Po H. Lu, George Bartzokis, Charles D. Smith, Greg Jicha, Peter Hardy, Partha Sinha, Elizabeth Oates, Gary Conrad, Neill R. Graf-Radford, Francine Parftt, Tracy Kendall, Heather Johnson, Oscar L. Lopez, MaryAnn Oakley, Donna M. Simpson, Martin R. Farlow, Ann Marie Hake, Brandy R. Matthews, Jared R. Brosch, Scott Herring, Cynthia Hunt, Anton P. Porsteinsson, Bonnie S. Goldstein, Kim Martin, Kelly M. Makino, M. Saleem Ismail, Connie Brand, Ruth A. Mulnard, Gaby Thai, Catherine Mc-Adams-Ortiz, Christopher H. van Dyck, Richard E. Carson, Martha G. MacAvoy, Pradeep Varma, Howard Chertkow, Howard Bergman, Chris Hosein, Sandra Black, Bojana Stefanovic, Curtis Caldwell, Ging-Yuek Robin Hsiung, Howard Feldman, Benita Mudge, Michele Assaly, Elizabeth Finger, Stephen Pasternack, Irina Rachisky, Dick Trost, Andrew Kertesz, Charles Bernick, Donna Munic, Kristine Lipowski, Masandra Weintraub, Borna Bonakdarpour, Diana Kerwin, Chuang-Kuo Wu, Nancy Johnson, Carl Sadowsky, Teresa Villena, Raymond Scott Turner, Kathleen Johnson, Brigid Reynolds, Reisa A. Sperling, Keith A. Johnson, Gad Marshall, Jerome Yesavage, Joy L. Taylor, Barton Lane, Allyson Rosen, Jared Tinklenberg, Marwan N. Sabbagh, Christine M. Belden, Sandra A. Jacobson, Sherye A. Sirrel, Neil Kowall, Ronald Killiany, Andrew E. Budson, Alexander Norbash, Patricia Lynn Johnson, Thomas O. Obisesan, Saba Wolday, Joanne Allard, Alan Lerner, Paula Ogrocki, Curtis Tatsuoka, Parianne Fatica, Evan Fletcher, Pauline Maillard, John Olichney, Owen Carmichael, Smita Kittur, Michael Borrie, T.-Y. Lee, Rob Bartha, Sterling Johnson, Sanjay Asthana, Cynthia M. Carlsson, Adrian Preda, Dana Nguyen, Pierre Tariot, Anna Burke, Nadira Trncic, Adam Fleisher, Stephanie Reeder, Vernice Bates, Horacio Capote, Michelle Rainka, Douglas W. Scharre, Maria Kataki, Anahita Adeli, Earl A. Zimmerman, Dzintra Celmins, Alice D. Brown, Godfrey D. Pearlson, Karen Blank, Karen Anderson, Laura A. Flashman, Marc Seltzer, Mary L. Hynes, Robert B. Santulli, Kaycee M. Sink, Leslie Gordineer, Jef D. Williamson, Pradeep Garg, Franklin Watkins, Brian R. Ott, Henry Querfurth, Geofrey Tremont, Stephen Salloway, Paul Malloy, Stephen Correia, Howard J. Rosen, Bruce L. Miller, David Perry, Jacobo Mintzer, Kenneth Spicer, David Bachman, Elizabether Finger, Stephen Pasternak, Irina Rachinsky, John Rogers, Dick Drost, Nunzio Pomara, Raymundo Hernando, Antero Sarrael, Susan K. Schultz, Laura L. Boles Ponto, Hyungsub Shim, Karen Ekstam Smith, Norman Relkin, Gloria Chaing, Michael Lin, Lisa Ravdin, Amanda Smith, Balebail Ashok Raj, Kristin Fargher

**Affiliations:** 1 School of Electrical Engineering and Computer Science, Gwangju Institute of Science and Technology, Gwangju, Korea; 2 UC San Francisco, San Francisco, CA 94107 USA; 3 UC San Diego, La Jolla, CA 92093 USA; 4 Mayo Clinic, Rochester, MN USA; 5 UC Berkeley, Berkeley, San Francisco USA; 6 University of Pennsylvania, Philadelphia, PA 19104 USA; 7 USC, Los Angeles, CA 90032 USA; 8 UC Davis, Sacramento, CA USA; 9 Brigham and Women’s Hospital/Harvard Medical School, Boston, MA 02215 USA; 10 Indiana University, Bloomington, IN 47405 USA; 11 Washington University St. Louis, St. Loui, MO 63110 USA; 12 Prevent Alzheimer’s Disease, Rockville, MD 20850 USA; 13 Siemens, Erlangen, Germany; 14 Alzheimer’s Association, Chicago, IL 60631 USA; 15 University of Pittsburg, Pittsburgh, PA 15213 USA; 16 Cornell University, Ithaca, NY 14853 USA; 17 Albert Einstein College of Medicine, Yeshiva University, Bronx, NY 10461 USA; 18 AD Drug Discovery Foundation, New York, NY 10019 USA; 19 Acumen Pharmaceuticals, Livermore, CA 94551 USA; 20 Northwestern University, Chicago, IL 60611 USA; 21 National Institute of Mental Health, Bethesda, MD 20892 USA; 22 Brown University, Providence, RI 02912 USA; 23 University of Washington, Seattle, WA 98195 USA; 24 University of London, London, UK; 25 UCLA, Torrance, CA 90509 USA; 26 University of Michigan, Ann Arbor, MI 48109-2800 USA; 27 University of Utah, Salt Lake City, UT 84112 USA; 28 Banner Alzheimer’s Institute, Phoenix, AZ 85006 USA; 29 UUC Irvine, Orange, CA 92868 USA; 30 Johns Hopkins University, Baltimore, MD 21205 USA; 31 Richard Frank Consulting, New York, USA; 32 National Institute on Aging, Baltimore, MD USA; 33 Oregon Health and Science University, Portland, OR 97239 USA; 34 University of Alabama, Birmingham, AL USA; 35 Mount Sinai School of Medicine, New York, NY USA; 36 Rush University Medical Center, Chicago, IL 60612 USA; 37 Baylor College of Medicine, Houston, TX USA; 38 Wien Center, Miami Beach, FL 33140 USA; 39 Columbia University Medical Center, New York, NY USA; 40 New York University, New York, NY USA; 41 University of Texas Southwestern Medical School, Galveston, TX 77555 USA; 42 Duke University Medical Center, Durham, NC USA; 43 Emory University, Atlanta, GA 30307 USA; 44 Medical Center, University of Kansas, Kansas City, KS USA; 45 University of Kentucky, Lexington, KY USA; 46 Mayo Clinic, Jacksonville, FL USA; 47 University of Rochester Medical Center, Rochester, NY 14642 USA; 48 Yale University School of Medicine, New Haven, CT USA; 49 McGill Univ. Montreal-Jewish General Hospital, Montreal, PQ H3A 2A7 Canada; 50 Sunnybrook Health Sciences, Toronto, ON Canada; 51 U.B.C. Clinic for AD & Related Disorders, Vancouver, BC Canada; 52 Cognitive Neurology - St. Joseph’s, London, ON Canada; 53 Cleveland Clinic Lou Ruvo Center for Brain Health, Las Vegas, NV 89106 USA; 54 Premiere Research Inst (Palm Beach Neurology), W Palm Beach, FL USA; 55 Georgetown University Medical Center, Washington, DC 20007 USA; 56 Stanford University, Stanford, CA 94305 USA; 57 Boston University, Boston, MA USA; 58 Howard University, Washington, DC 20059 USA; 59 Case Western Reserve University, Cleveland, OH 44106 USA; 60 Neurological Care of CNY, Liverpool, NY 13088 USA; 61 St. Joseph’s Health Care, London, ON N6A 4H1 Canada; 62 Dent Neurologic Institute, Amherst, NY 14226 USA; 63 Ohio State University, Columbus, OH 43210 USA; 64 Albany Medical College, Albany, NY 12208 USA; 65 Hartford Hospital Olin Neuropsychiatry Research Center, Hartford, CT 06114 USA; 66 Dartmouth-Hitchcock Medical Center, Lebanon, NH USA; 67 Wake Forest University Health Sciences, Winston-Salem, NC USA; 68 Medical University South Carolina, Charleston, SC 29425 USA; 69 Nathan Kline Institute, Orangeburg, NY USA; 70 University of Iowa College of Medicine, Iowa City, IA 52242 USA; 71 USF Health Byrd Alzheimer’s Institute, University of South Florida, Tampa, FL 33613 USA

**Keywords:** Alzheimer's disease, Transcription

## Abstract

Alzheimer’s disease (AD) is a complex and heterogeneous disease that can be affected by various genetic factors. Although the cause of AD is not yet known and there is no treatment to cure this disease, its progression can be delayed. AD has recently been recognized as a brain-specific type of diabetes called type 3 diabetes. Several studies have shown that people with type 2 diabetes (T2D) have a higher risk of developing AD. Therefore, it is important to identify subgroups of patients with AD that may be more likely to be associated with T2D. We here describe a new approach to identify the correlation between AD and T2D at the genetic level. Subgroups of AD and T2D were each generated using a non-negative matrix factorization (NMF) approach, which generated clusters containing subsets of genes and samples. In the gene cluster that was generated by conventional gene clustering method from NMF, we selected genes with significant differences in the corresponding sample cluster by Kruskal–Wallis and Dunn-test. Subsequently, we extracted differentially expressed gene (DEG) subgroups, and candidate genes with the same regulation direction can be extracted at the intersection of two disease DEG subgroups. Finally, we identified 241 candidate genes that represent common features related to both AD and T2D, and based on pathway analysis we propose that these genes play a role in the common pathological features of AD and T2D. Moreover, in the prediction of AD using logistic regression analysis with an independent AD dataset, the candidate genes obtained better prediction performance than DEGs. In conclusion, our study revealed a subgroup of patients with AD that are associated with T2D and candidate genes associated between AD and T2D, which can help in providing personalized and suitable treatments.

## Introduction

The number of people worldwide suffering from Alzheimer’s disease (AD) has been steadily increasing in recent decades^1^. AD is an irreversible disease that slowly and progressively destroys the brain. Specifically, AD affects memory dysfunction, representing a major cause of dementia in the aging population^2,3^. However, AD is not a normal component of aging, but is instead a complex disease entity, and the detailed pathogenic mechanisms underlying the disease remain unclear. It has been generally recognized that accumulation of plaques (beta-amyloid) and tangles (tau) is the leading cause of AD^4^, and the prime suspect contributing to the associated neuronal destruction^5,6^. Although the specific causes of beta-amyloid and tau accumulation are unknown, this pathogenic event is considered to be the result of various interacting genetic and environmental factors^7^. Therefore, it is important to address the complexity of AD by detecting the underlying characteristics.

One approach to disentangle a complex disease is gene expression analysis, including the identification of potential candidate genes or comparing expression values for specific AD-related genes. Indeed, several studies have discovered AD-related genes and mechanisms using genome-wide analyses^8–11^. In particular, AD analyses using blood samples from patients have received considerable attention as a novel method of diagnosis given the advantages of the non-invasive nature and less expensive process compared to traditional analyses using brain tissue or imaging. In fact, several differentially expressed proteins in the AD brain have been identified in the blood of AD patients^12^. Bu et al.^13^ showed that the amyloid-beta protein is produced not only in the brain, but also in the peripheral tissues of AD patients. Therefore, many studies have focused on machine-learning and statistical methods to improve early detection and for the identification of candidate genes that can serve as targets for AD treatment based on data obtained from peripheral samples^14–17^.

Moreover, recent studies have indicated an association of AD with other diseases^18–20^. Several genetic factors and pathophysiological mechanisms associated with AD are also shared with other AD-related diseases^21^. Therefore, identifying the relationships between AD and AD-related diseases can help to address the complexity of AD and bring us a step closer to realizing the personalized treatment of AD by reducing risk factors from comorbidities and giving a chance to treat common dysregulated pathways between AD and it related diseases^22,23^. Narayanan et al.^24^ investigated co-regulated genes in AD and Huntington’s disease, and identified common differentially co-expressed subnetworks in the two neurodegenerative diseases. In particular, type 2 diabetes (T2D) is highly associated with AD, and epidemiological studies have shown that patients with T2D have a higher risk of developing AD^25^. One of the hallmark pathophysiologic features in T2D patients is the deposition of amyloid converted from islet amyloid peptide (IAPP)^26^. Overproduction of IAPP secreted by pancreatic beta-cells may cause beta cell loss in T2D, and there are evidences that intracellular toxic amyloid peptide oligomers are associated with AD^27^. Given that several lines of evidence indicate a link between AD and T2D, AD can be considered as a brain-specific type of diabetes that has been dubbed “type 3 diabetes”, and inflammation, insulin resistance, and mitochondrial dysfunction were considered as common pathogenesis of two diseases^28–30^.

**Figure 1:**
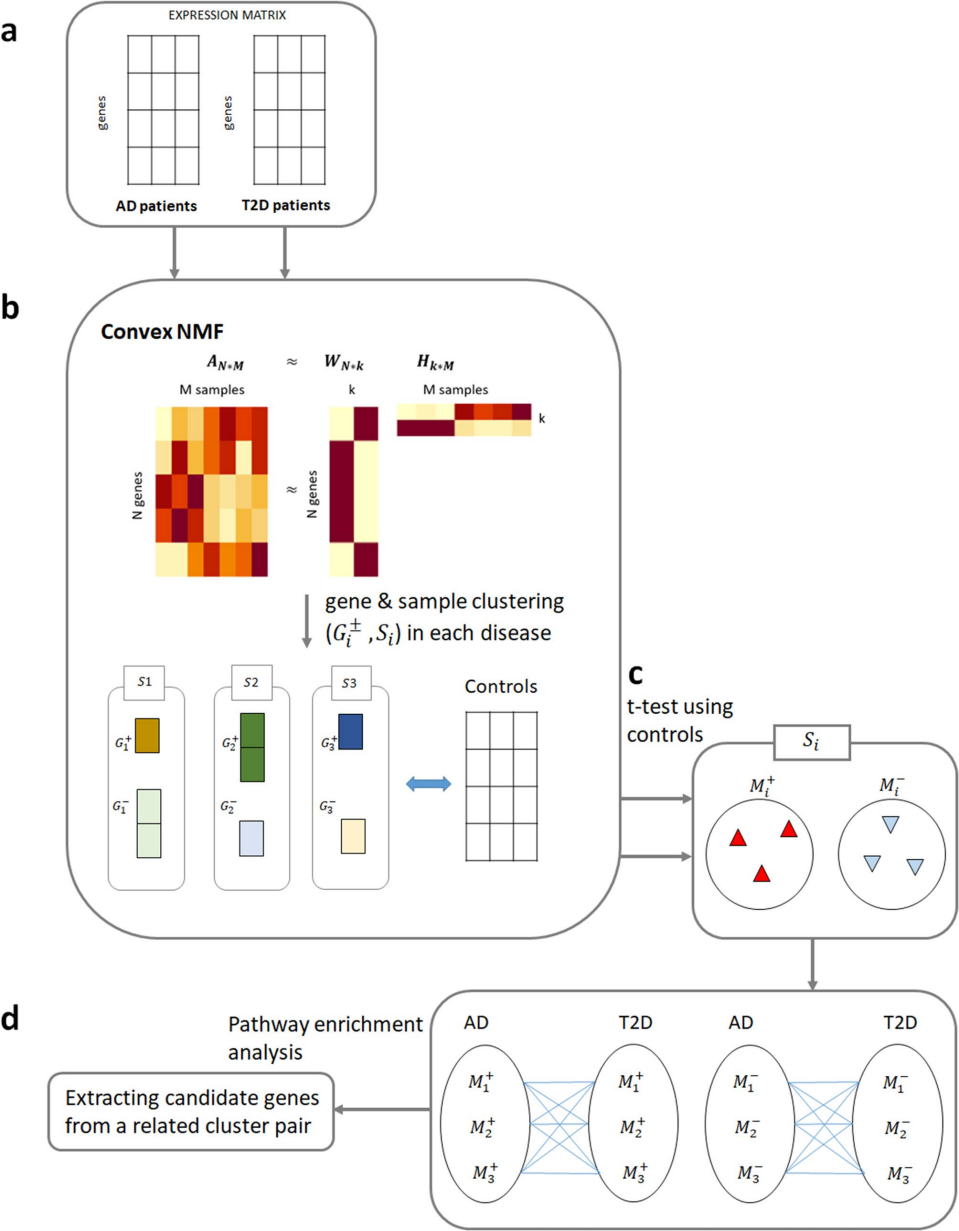
Procedure for relation extraction between two diseases. (**a**) Gene expression data of AD and T2D patients are given. (**b**) The convex non-negative matrix factorization (NMF) is used to decompose the input expression matrices. Gene and sample clusters are obtained from the NMF decomposed matrices. (**c**) DEG genes are assigned to clusters. (**d**) Common candidate genes are extracted from a related cluster pair between two diseases. (**b**) is generated by the R software (R version 3.6.1, https://www.r-project.org/) using an example dataset.

Accordingly, the aim of this study was to detect the common and distinct characteristics of AD and T2D using gene expression datasets. Because not all AD patients show an association with T2D, we extracted genes with significantly different expression profiles in AD and T2D patients separately using computational and statistical methods, and then defined subgroups of AD and T2D compared to their respective controls. Based on this analysis, we identified common genes with the same regulation directions (up or downregulated in the disease) in each pair of patient subgroups. Through this approach, we identified candidate genes among the common genes with significant differences in expression levels in the subgroups of the two diseases, which were tested as potential biomarkers for diagnostic or prognostic prediction using an independent Alzheimer’s Disease Neuroimaging Initiative (ADNI) dataset^31^. Figure 1 illustrates the procedure for relation extraction between two diseases.

## Methods

### Data description

The mRNA expression datasets for AD and T2D were downloaded from the Gene Expression Omnibus (GEO) (https://www.ncbi.nlm.nih.gov/geo/). For AD analysis, we used integrated data on peripheral blood gene expression profiles from the GSE63060 and GSE63061 datasets using an R software^32^, which were generated on the Illumina HumanHT-12 v3.0 Expression BeadChip and the Illumina HumanHT-12 v4.0 Expression BeadChip, respectively. The GSE63060 dataset contains data for 145 AD patients and 104 control samples, and the GSE63061 dataset contains data for 140 AD patients and 135 control samples. For T2D, we used gene expression data (GSE78721) including 68 T2D patients and 62 healthy controls from adipocytes and infiltration macrophages because it was the largest T2D dataset in GEO, and adipose-derived transcription signature is associated with T2D^33,34^. Gene expression data were generated using the Affymetrix PrimeView Human Gene Expression Array.

For each gene expression data point from the GEO datasets, the probe ID was converted into the Entrez Gene ID using information from the platforms of each AD and T2D datasets (e.g., GPL6947, GPL10558, and GPL15207). Gene expression levels of the probe that did not match the Entrez Gene ID were removed. Among all the assigned Entrez Gene IDs, protein-coding genes were selected using database Homo_sapiens.GRCh38.94 (http://asia.ensembl.org/Homo_sapiens/Info/Index), where Ensembl IDs were converted to the Entrez Gene IDs using the “biomaRt” package in R software. For duplicated Entrez Gene IDs, the expression values of the same Entrez Gene ID were merged into the mean value. To analyze the AD data, we merged the two datasets (GSE63060 and GSE63061). The number of Entrez Gene IDs selected by the protein-coding gene database in each AD dataset was 16,730 and 14,957, respectively. We selected 14,134 common genes from the two datasets and used the “removeBatchEffect” function in the R package “limma”. As a result, we obtained the expression data of 14,134 genes from the 285 AD patients and 239 control samples. In addition, we normalized each data point in the patient expression data by applying a log2(fold change) conversion as follows:

$$\begin{aligned} y_{ij} = \log _{2}{\frac{patient_{ij}}{normal_i}} \end{aligned}$$

where $patient_{ij}$ is the expression value of the gene *i* of the *j*th patient and $normal_i$ is the mean expression value of the gene *i* in the normal control samples.

### Decomposition of gene expression data sets

Most gene expression datasets contain information on thousands of genes, which is relatively large compared to the number of samples; therefore, several studies have applied dimensionality reduction methods to reduce the matrix dimension^35,36^. The non-negative matrix factorization (NMF) method has been widely used to reduce the dimension of an input matrix by decomposing a non-negative input matrix into two matrices^37^. Assuming an input matrix *A* consisting of the expression data of *N* genes and *M* samples, NMF produces the matrices *W* and *H* of size $N\times k$ and $k\times M$, respectively, in which the parameter *k* indicates the number of clusters desired in the input data. The NMF algorithm is a multiplication update algorithm that multiplies *W* and *H* to obtain the input *A* during iteration until convergence. After convergence, the matrices *W* and *H* are used to bi-cluster genes and samples^38,39^. Each row of $W (gene\times k)$ and each column of $H (k\times sample)$ can be represented by a positive linear combination of *k*. The element $w_{ij}$ in the *W* matrix is the coefficient of gene *i* and cluster $j (1\sim k)$, and the element $h_{ij}$ in the *H* matrix is the coefficient of cluster $i (1\sim k)$ and sample *j* (Fig. 1b).

### Determination of the optimal number of clusters according to the rank k

To select the optimal number of meaningful clusters that correctly divide the input data, the cophenetic correlation coefficient should be taken into consideration^37^. NMF updates the $W_i$ and $H_i$ matrices of each *i*th iteration until convergence. Following this, a connectivity matrix $C_i$ (with size $sample\times sample$) is defined by each sample assignment of the *i*th iteration from $H_i$ by selecting the maximum index of each column. The elements of the connectivity matrix $c_{ij}$ are filled with 1 s if the samples *i* and *j* are assigned to the same cluster and are filled with 0 s otherwise. The average of all connectivity matrices represents the consensus matrix ${\bar{C}}$, which is the probability that the samples *i* and *j* belong together during iterations. The cophenetic correlation coefficient is then calculated as the Pearson correlation coefficient between $I-{\bar{C}}$ and the distance between samples in a hierarchical clustering of ${\bar{C}}$. The cophenetic correlation coefficient indicates the dispersion of the sample assignment, which refers to how consistently samples with similar gene expression profiles belong together during iterations. Therefore, the rank *k* with the highest cophenetic correlation indicates the optimal capacity of the model.

However, NMF can only process non-negative ranges of entries in the input matrix *A*, and the output matrices *W* and *H* also have non-negative ranges. Therefore, to analyze the log2(fold change) expression dataset of each disease, we needed to select a model that can cover both positive and negative values. If the range of the input matrix *A* is ${\pm }$, then the matrix $A_{\pm }$ is decomposed such that $A_{\pm } \approx W_{\pm }\times H_+$. In convex NMF, the basis vectors of the $W_{\pm }$ matrix are considered to be convex combinations of the input matrix $A_{\pm }$ (i.e., $A_{\pm } \approx W_{\pm }\times H_+ \approx X_{\pm }\times F_+ \times H_+$^40^) and there is an advantage in that the factors of $F_+$ and $H_+$ are sparse. Therefore, we apply the convex NMF to obtain the *W* and *H* matrices. $X_{\pm }\times F_+$ from convex NMF is treated as a factor of W in the NMF used for gene clustering, and then $F_+$ and $H_+$ are updated alternatively as follows:

$$\begin{aligned} H_{ik} \leftarrow H_{ik} \sqrt{\frac{[(X^TX)^+F]_{ik}+ [HF^T(X^TX)^-F]_{ik}}{[(X^TX)^-F]_{ik} + [HF^T(X^TX)^+F]_{ik}}} \\ F_{ik} \leftarrow F_{ik}\sqrt{\frac{[(X^TX)^+H]_{ik} + [(X^TX)^-FH^TH]_{ik}}{[(X^TX)^-H]_{ik} + [(X^TX)^+FH^TH]_{ik}}} \end{aligned}$$

### Gene and sample clusters

The basic method of gene and sample clustering in NMF using the factors *W* and *H* is the “Max” method, which selects the cluster with the highest coefficient^37^. In general, a gene is assigned to the cluster with the highest coefficient in each gene row in the *W* matrix, and a sample is assigned to cluster $S_i$ when the *i*th coefficient is the highest coefficient in each sample column in the *H* matrix. Accordingly, the gene cluster obtained by the “Max” gene clustering method is a group of genes with relatively upregulated expression in the sample cluster $S_i$ compared to other sample clusters. Conversely, to consider the gene cluster with relatively downregulated expression, a gene is assigned to the cluster with the minimum coefficient using the “Min” gene clustering method. We performed this bi-clustering method via NMF using the AD patient expression data to cluster AD patients into *k* sample clusters and genes into *k* relatively up and downregulated clusters, respectively. The T2D patient expression data were processed in the same manner.

However, in most gene expression analyses, the number of genes (features) is larger than the number of samples in the dataset. Even if the dimension of genes and samples in an expression dataset can be reduced using NMF, it is still difficult to analyze genes in *k* clusters because each gene belongs to one of the *k* clusters. In addition, each cluster may contain genes whose expression values in the samples of the given cluster are not different compared with those in samples of other clusters. Thus, some genes will be assigned to a gene cluster even if there are no relative differences in expression between sample clusters (Supplementary Fig. S1).

To address this issue, we filtered out genes in clusters by considering the original input matrix *A* and identified which genes in each cluster showed significantly different expression levels in a specific sample cluster. First, for each gene that was already assigned to the cluster, the distribution of expression levels was defined as $D_i (i=1\sim k)$ for each *k* sample cluster. We then used the Kruskal–Wallis test to identify genes with a significantly different expression distribution in samples of a given cluster from those in samples of at least one other cluster. The *p* values of the test were adjusted according to the Bonferroni correction for multiple comparisons, and genes with a *q* value < 0.05 were selected. Further, the Dunn test was performed between the distribution of expression levels for each gene in all possible sample cluster pairs. If the expression level differences of the gene between the given cluster and other remaining clusters were significant (*q* values < 0.05 after Bonferroni correction), the gene was selected. For cluster *i*, these genes with relatively upregulated expression were denoted as $G_i^+$ (Fig. 1b).

Similarly, for a gene assigned to the gene cluster by the “Min” gene clustering method, the Kruskal–Wallis test and Dunn test were subsequently applied to the genes in each cluster. For cluster *i*, these genes with relatively downregulated expression were selected and denoted as $G_i^-$. This process could effectively reduce the number of genes in each cluster compared to the conventional clustering method of NMF, which facilitated the analysis.

### Differentially expressed gene (DEG) subgroups

The Kruskal–Wallis–based gene clustering method described above can extract the genes with relatively up and downregulated expression in patients with a given disease in *k* sample clusters. However, we further needed to identify whether genes in the obtained $G_i^+$ and $G_i^-$ groups are differentially expressed in the sample cluster $S_i$ compared to controls. In addition, even if $G_i$ selected as the characteristic of $S_i$ differs significantly from other sample clusters, genes in $G_i$ need to be differentially expressed in $S_j$ compared to controls, which means that it can also be the characteristic of the $S_j$ sample cluster. Especially, because AD and T2D datasets are gene expression data from different tissue types, by extracting differentially expressed genes between each patient groups of AD and T2D and their healthy controls, we can remove tissue-specific genes and detect genes related to each disease. Therefore, we considered DEGs from all genes in $G_i^+$ and $G_i^-$ between each patient cluster and their respective controls. First, we collected all genes assigned to any *k* cluster. Second, the expression levels for each gene were compared between disease samples in the *i* cluster and control samples using the *t*-test followed by Bonferroni correction. The genes with a *q* value < 0.05 for the sample *i* cluster were assigned to $M_i^+$ or $M_i^-$ depending on the direction of the expression level difference (upregulation or downregulation, respectively) (Fig. 1c).

Because AD and T2D gene expression datasets were obtained from different tissue types, this subgrouping of genes based on DEG is necessary. We can remove tissue-specific genes and select genes related to each disease by using DEGs between each patient groups of AD and T2D and their healthy controls.

### Extraction of AD and T2D-related subgroup pairs and candidate genes

We independently applied the NMF approach for the expression data of AD and T2D patients and their respective controls, and obtained AD and T2D DEG subgroups for specific sample clusters. We then aimed to find AD subgroups related to T2D and T2D subgroups related to AD. To this end, we performed pathway enrichment analysis. These enriched pathways were then compared with those of known AD- and T2D-related genes in DigSee^41^. In addition, we investigated whether the enriched pathways in the AD DEG subgroups overlapped with T2D-related pathways and vice versa. Then, we selected a AD subgroup and a T2D subgroup containing the largest number of T2D-related pathways and AD-related pathways, respectively, which are a pair of clusters related with each other. From these two clusters, we selected common candidate genes with the same regulation direction, which are referred to as candidate genes (Fig. 1d).

Afterwards, we used an independent AD dataset downloaded from the ADNI (http://adni.loni.usc.edu)^31^, which included gene expression data from 116 AD patients and 246 controls extracted from peripheral blood, to validate the candidate genes identified from the DEG subgroup pairs related to both the diseases. With this dataset, the expression levels of 20,384 protein-coding genes filtered using the database Homo sapiens.GRCh38.94 were used for the classification of AD and the control sample using logistic regression. Tenfold cross-validation was performed with zero initialization and a learning rate of 0.05, and the area under the curve (AUC) was calculated at each tenfold cross-validation to evaluate the predictive ability of the candidate genes.

Additionally, we collected independent T2D gene expression datasets: 25 T2D patient and 71 control samples extracted from beta-cells or pancreatic islets in GSE20966, GSE25724, and GSE38642^42–45^. Then, we selected protein-coding genes using Homo sapiens.GRCh38.94 from each dataset. By removing the batch effect using the “removeBatchEffect” function in the R package “limma”, we normalized the expression profiles for 10,490 common genes in the three datasets and merged them. Similar to AD, we validated the candidate genes using these T2D datasets. Because of the small number of T2D patients, we performed threefold cross-validation with zero initialization and a learning rate of 0.005 in a logistic regression model.

## Results and discussion

### Clustering of AD and T2D genes

**Figure 2:**
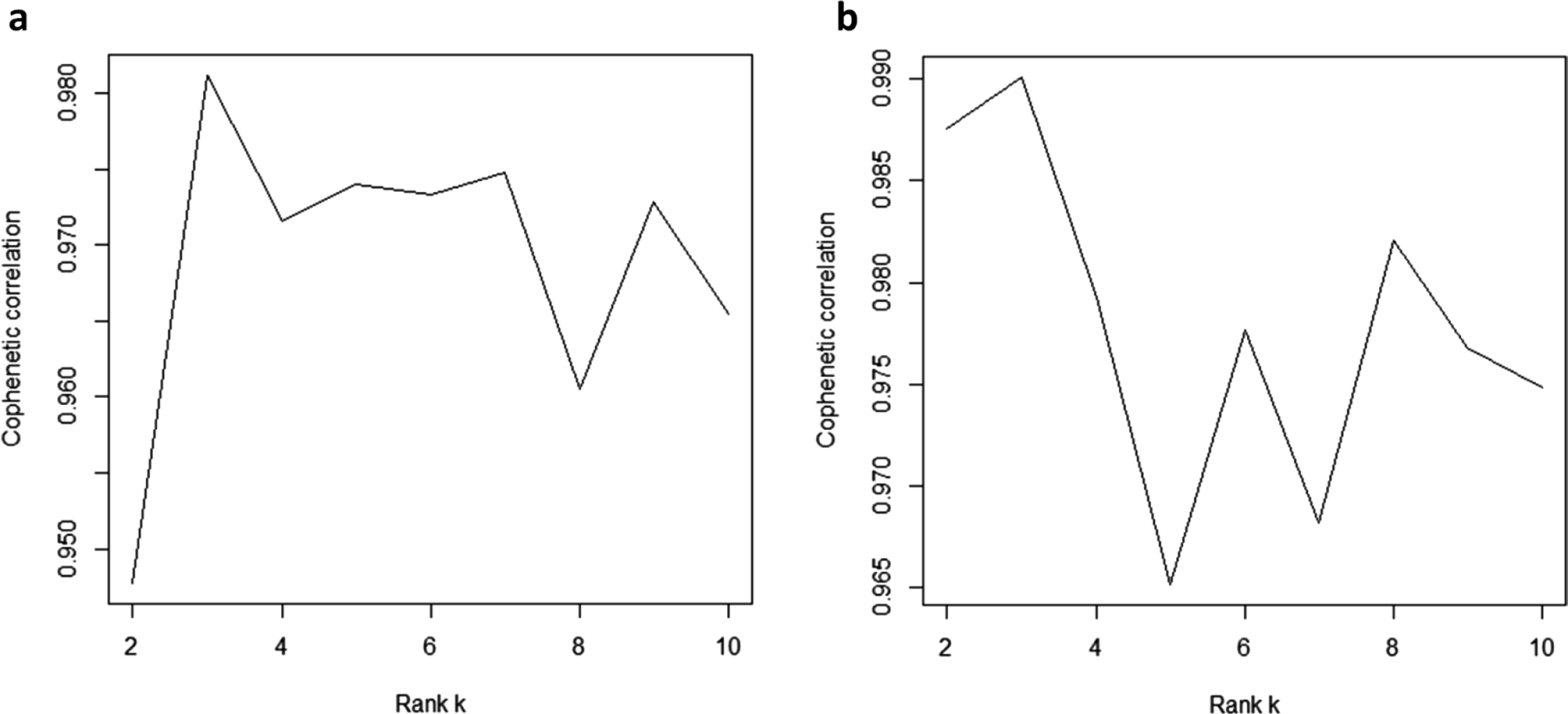
Cophenetic correlation coefficients for the consensus matrix in (**a**) Alzheimer’s disease (AD) and (**b**) type 2 diabetes (T2D) datasets.

The AD and T2D subgroups were independently defined using the log2(fold change) values from the expression data of 285 AD and 68 T2D patients using the convex NMF approach and NMF-based clustering method. First, to decompose the expression data of the patients into subgroups using the NMF approach, we needed to determine the optimal number of subgroups. In general, initialization of the matrices *W* and *H* affects the final outputs of NMF. Hence, we applied the NMF algorithm 10 times for each rank *k* from 2 to 10 with randomly initialized *W* and *H* matrices, and then calculated the average of the cophenetic correlation coefficient. We chose the rank *k* that had the largest average cophenetic correlation coefficient. Figure 2 shows the average cophenetic correlation coefficients for each rank *k* in each AD and T2D patient dataset. The optimal rank *k* of both datasets was 3. Thus, we used the factorized matrices *W* and *H* with the largest cophenetic correlation coefficient out of 10 iterations of rank 3 as the NMF output for each disease.

After applying the sample and gene assignment method to the decomposed matrices, we constructed three clusters containing a subset of samples for genes with relatively upregulated and downregulated expression, respectively. For sample clustering, the “Max” method was applied to matrix *H* in columns, and the 285 AD patients and 68 T2D patients were divided into three sample clusters of 93, 90, and 102 patients, and 17, 9, and 42 patients, respectively. All of the genes in both datasets were first assigned to one of the three gene clusters through the “Max” method. According to the Kruskal-Wallis and Dunn test, 5375 and 4479 genes were significantly upregulated ($G_x^+$) and downregulated ($G_y^-$), respectively, in expression from other clusters for AD. Similarly, 3461 and 7369 genes were upregulated and downregulated, respectively, for T2D (Table 1). Each gene can be assigned to both $G_x^+$ and $G_y^-$ when the distribution in $S_x$ is relatively upregulated whereas that in $S_y$ is relatively downregulated. Thus, in the union of upregulated and downregulated genes, 6729 and 10,051 genes emerged as showing significant differences in expression from other clusters for AD and T2D, respectively.

Each gene can be assigned to both $G_x^+$ and $G_y^-$ when the distribution in $S_x$ is relatively upregulated whereas that in $S_y$ is relatively downregulated. The Kruskal-Wallis based gene clustering method showed that samples and genes in AD patients were more evenly divided compared to those in T2D patients. However, in the T2D dataset, most of the samples were assigned to $S_3$ and the gene cluster also showed a skewed distribution in $G_3$. Because the number of clusters was decided by the optimal rank *k*, the cluster $S_2$ with the small number samples can be generated when the total number of samples are small such as the T2D dataset. This small size cluster may have distinct characteristics that can be distinguished from other clusters.

**Table 1:** Clustered samples and genes for (a) Alzheimer’s disease (AD) and (b) type 2 diabetes (T2D).

**(a)** AD			
Cluster *i*	$S_i$	$G_i^+$	$G_i^-$
1	93	3004	1989
2	90	1242	1742
3	102	1129	748

**(b)** T2D			
Cluster *i*	$S_i$	$G_i^+$	$G_i^-$
1	17	116	144
2	9	302	564
3	42	3043	6661

**Figure 3:**
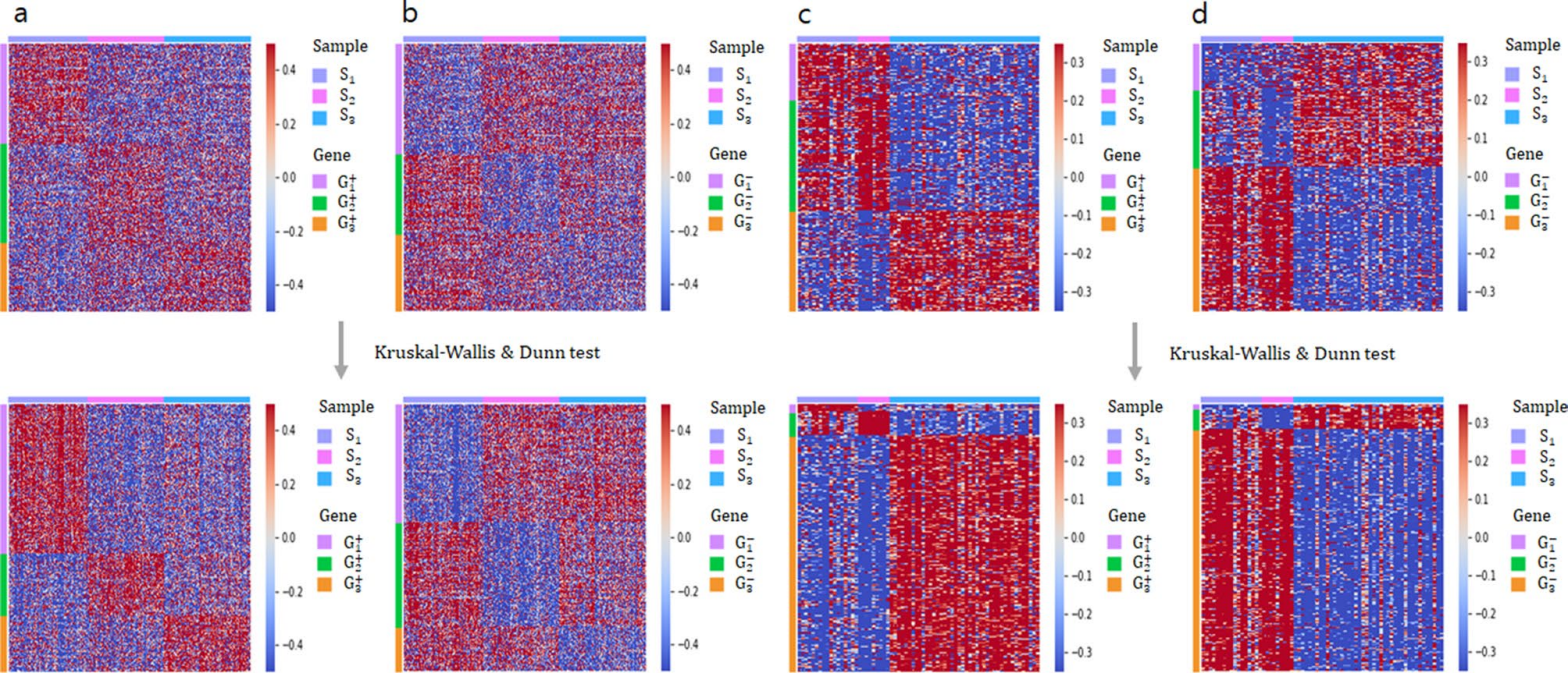
Heatmap of gene clustering method in Alzheimer’s disease (AD) and type 2 diabetes (T2D). (**a**) and (**b**) show genes and sample clusters from AD patients. (**c**) and (**d**) show genes and sample clusters from T2D patients. Top panels show clusters obtained using the basic “Max” ((**a**) and (**c**)) and “Min” ((**b**) and (**d**)) methods. Bottom panels show clusters obtained using the Kruskal–Wallis and Denn test for relatively upregulated genes ((**a**) and (**c**)) and relatively downregulated genes ((**b**) and (**d**)). This figure is generated by the R software (R version 3.6.1, https://www.r-project.org/) and python (version 3.6.8, https://www.python.org/).

To visually confirm that the Kruskal-Wallis–based gene clustering method removed inappropriate genes in each gene cluster compared to the conventional “Max”/“Min” method, the gene expression matrices were rearranged in cluster order for both genes and samples, which were visualized on a heatmap. The rectangular regions on the diagonal of the heatmap, indicating samples and genes assigned in the same cluster, demonstrate genes with relatively upregulated or downregulated expression in each cluster. Compared to the basic “Max” and “Min” method, genes selected by Kruskal-Wallis test generated more distinct regions, in which genes showed significant differences in expression levels that could be clearly observed on the heatmap for both the AD and T2D datasets (Fig. 3). Figure 3 was generated using “aheatmap” function in the R package “NMF”^46^ and “heatmap” function in the python library “seaborn”^47^.

Next, we constructed $M_i^+$ and $M_i^-$, where gene expressions differ between each disease patient group and its corresponding control group. For the subgroups of each sample cluster, we integrated the genes with relatively up and downregulated expression compared to patients in other clusters ($G_i^+$ and $G_i^-$), resulting in 6729 and 10,051 integrated genes in AD and T2D, respectively. Then, genes were assigned into a subgroup *i* if they are differentially expressed between patients in the subgroup *i* and control samples based on the *t*-test with Bonferroni-corrected *q* value < 0.05. The numbers of these up and downregulated genes ($M_i^+$ and $M_i^-$) are shown in Table 2.

We further examined the clinical characteristics such as age of these samples in AD modules because the age information is only in AD, not T2D. The result of a one-way ANOVA for age in the AD patient subgroup showed no significant differences between AD patient subgroups (Supplementary Table S2), which suggests that the grouping of samples does not depend on age.

**Table 2:** Number of DEGs in each cluster.

**(a)** AD			
Cluster *i*	$S_i$	$M_i^+$	$M_i^-$
1	93	1281	825
2	90	1443	2422
3	102	1482	1670

**(b)** T2D			
Cluster *i*	$S_i$	$M_i^+$	$M_i^-$
1	17	228	426
2	9	41	283
3	42	1235	4113

### Selecting cluster pairs of interest

To find AD clusters related to T2D and T2D clusters related to AD, pathway enrichment analysis was performed for the up and downregulated genes ($M_i^+$ and $M_i^-$) in each AD and T2D cluster using a total of 10,378 Kyoto Encyclopedia of Genes and Genomes (KEGG) pathways and Gene Ontology (GO) terms from MSigDB. The hypergeometric test for $M_i^+$ and $M_i^-$ in AD and T2D genes was used to determine significantly enriched KEGG pathways and GO terms (Tables 3 and 4). As references of AD- and T2D-related pathways, we extracted 1635 AD-related genes and 1658 T2D-related genes from DigSee^41^, and obtained 1675 and 1757 AD- and T2D-related pathways, respectively, using the hypergeometric enrichment test. We identified common pathways from the DEG subgroups in AD patients with 1757 T2D-related pathways from DigSee^41^. Among the AD clusters, patients in $S_3$ were most likely to have an association with T2D compared to AD patients in the AD $S_1$ and AD $S_2$ clusters (Table 3). Likewise, T2D patients in cluster $S_3$ were most likely to be associated with AD (Table 4). There was no clinical information in the datasets to confirm whether the AD $S_3$ patients actually have T2D or whether the T2D $S_3$ patients have AD; however, our data suggest that patients in these clusters might share genetic characteristics of the other disease.

In addition, we used brain gene expression data GSE5281 to determine which AD clusters have features in common with the AD brain^48^. We extracted 1831 DEGs from these gene expression data by performing the *t*-test with a *q* value < 0.05, and 160 pathways were extracted from these genes. As a result, genes in the AD $S_3$ module most overlapped with AD brain-related pathways (Supplementary Table S1).

**Table 3:** Numbers of type 2 diabetes (T2D)-related pathways for each Alzheimer’s disease (AD) differentially expressed gene module.

AD $M_i$	Enriched pathways	Common pathways with 1757 T2D-related pathways
$M_1^+$	16	0
$M_1^-$	10	5
$M_2^+$	30	9
$M_2^-$	101	4
$M_3^+$	205	119
$M_3^-$	164	8

**Table 4:** Numbers of Alzheimer’s disease (AD)-related pathways in each type 2 diabetes (T2D) differentially expressed gene module.

T2D $M_i$	Enriched pathways	Common pathways with 1675 AD-related pathways
$M_1^+$	4	0
$M_1^-$	5	5
$M_2^+$	0	0
$M_2^-$	0	0
$M_3^+$	135	31
$M_3^-$	87	16

To obtain further evidence that AD $S_3$ and T2D $S_3$ are related, pathway enrichment analysis was performed for the common genes of possible cluster pairs in the up-regulated and down-regulated AD and T2D gene clusters. The common genes with the same regulation direction (i.e., up or downregulated) in both diseases were extracted, and their enriched pathways were compared with the 1498 pathways of the 671 intersecting genes between AD and T2D from DigSee^41^. We used the pathways associated with both AD and T2D as references to identify whether the common genes in each possible cluster pair were related. Indeed, the enriched pathways from common genes in the upregulated AD $M_3^+$ and T2D $M_3^+$ modules showed more overlap with pathways from DigSee compared to that in other possible pairs (Table 5). Similarly, the common genes from the downregulated AD $M_3^-$ and T2D $M_3^-$ modules showed the highest overlapping pathway ratio with pathways from DigSee (Table 5). This suggested that the cluster pairs AD $M_3$ and T2D $M_3$ were the most closely related to both AD and T2D.

**Table 5:** Numbers of common pathways between disease subgroup pairs and DigSee.

AD $M_i$	T2D $M_i$	Pathways from common genes	Common pathways with DigSee
$M_1^+$	$M_1^+$	0	0
$M_1^-$	$M_1^-$	2	0
$M_1^+$	$M_2^+$	0	0
$M_1^-$	$M_2^-$	0	0
$M_1^+$	$M_3^+$	0	0
$M_1^-$	$M_3^-$	0	0
$M_2^+$	$M_1^+$	0	0
$M_2^-$	$M_1^-$	0	0
$M_2^+$	$M_2^+$	0	0
$M_2^-$	$M_2^-$	0	0
$M_2^+$	$M_3^+$	23	3
$M_2^-$	$M_3^-$	0	0
$M_3^+$	$M_1^+$	0	0
$M_3^-$	$M_1^-$	0	0
$M_3^+$	$M_2^+$	0	0
$M_3^-$	$M_2^-$	1	0
$M_3^+$	$M_3^+$	21	12
$M_3^-$	$M_3^-$	18	7

### Extraction of candidate genes

We extracted 241 common genes from the cluster pair AD $M_3$ and T2D $M_3$, including 195 upregulated genes and 46 downregulated genes, which were selected as candidate genes associated with both AD and T2D (Supplementary Table S3). Among these 241 genes, 14 genes were common with genes related with both AD and T2D from DigSee^41^. In DigSee, 661 genes were common for both AD and T2D. With a hypergeometric test for significance of 14 genes out of 241, a *p* value was 0.03826, showing significance of these genes in their roles in AD and T2D. In addition, we collected more AD and T2D genes from AlzGene and T2DiACoD (Supplementary Table S3)^49,50^. When comparing the 241 genes with genes in the three databases of DigSee, AlzGene, and T2DiACoD, 56 genes were related to AD or T2D.

These candidate genes were enriched in 29 pathways (Supplementary Table S4) and 14 pathways that are common with AD and T2D-related pathways from DigSee are shown in Table 6. Pathways associated with common pathological features of AD and T2D such as the immune system-related pathways (T cell selection, positive T cell selection, and T cell differentiation)^51,52^ and chemokine signaling pathway^53,54^ were included. Immune system-related pathways are known to be common characteristics of AD in the brain and blood^51^, and there are some evidences that chemokines play an essential role in the central nervous system and neuroprotection^55,56^. Interestingly, 11 genes among the 241 candidate genes were involved in chemokine signaling pathway, and 6 of them were AD and T2D-related genes: RAF1, RAC1, RHOA, STAT3, AKT1, and PRKCD.

**Table 6:** Common pathways between candidate genes and DigSee genes.

Pathways	*P* value	Adjusted *p* value
GO: Positive regulation of cell-cell adhesion	3.79E−09	2.85E−05
KEGG: Pancreatic cancer	2.17E-07	4.03E−05
GO: T cell selection	5.83E-09	4.39E−05
GO: Positive T cell selection	7.33E−09	5.52E−05
GO: T cell differentiation	5.64E−08	0.000425056
KEGG: Adherens junction	3.57E−06	0.000663158
KEGG: Epithelial cell signaling in Helicobacter pylori infection	2.23E−05	0.004142846
KEGG: Chemokine signaling pathway	2.58E−05	0.004806622
KEGG: JAK-STAT signaling pathway	2.62E−05	0.004876146
GO: Vacuolar lumen	1.12E−05	0.011236912
KEGG: T cell receptor signaling pathway	6.41E−05	0.011931807
KEGG: Colorectal cancer	0.000123344	0.022942068
KEGG: Neurotrophin signaling pathway	0.000188801	0.035116995
KEGG: Fc gamma R-mediated phagocytosis	0.00020364	0.037877102

To verify whether the 241 candidate genes could be informative markers for the classification of each disease patients and controls, we used data from the ADNI cohort^31^ for AD prediction, and a merged independent T2D dataset for predicting T2D. In AD prediction, candidate genes from the (AD $M_3$, T2D $M_3$) pair showed the best diagnostic performance, with an AUC value of 0.6906, compared with genes from nine possible pairs (Table 7).

**Table 7:** Performance of classification of Alzheimer’s disease (AD) and controls in the ADNI cohort using different sets of genes. ($M_i,M_j$) represents the common genes between the AD $M_i$ module and type 2 diabetes (T2D) $M_j$ module used for classification.

(AD $M_i$, T2D $M_j$)	AUC
($M_1,M_1$)	0.5173
($M_1,M_2$)	0.6034
($M_1,M_3$)	0.5810
($M_2,M_1$)	0.5264
($M_2,M_2$)	0.5763
($M_2,M_3$)	0.6256
($M_3,M_1$)	0.5411
($M_3,M_2$)	0.6022
($M_3,M_3$)	0.6906

**Figure 4:**
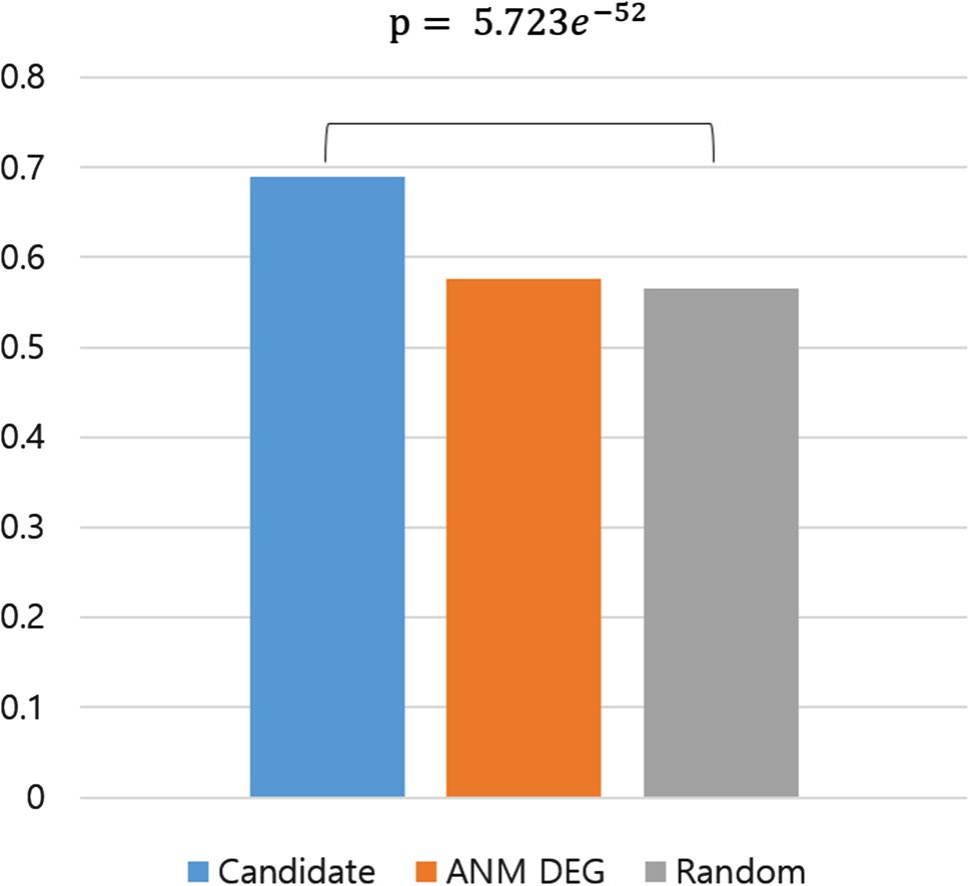
Performance comparison of Alzheimer’s disease (AD) classification from controls based on the area under the curve (AUC) using different gene sets.

For comparison, the performance of classifying AD in the ADNI cohort was measured for 250 random genes to match the size of the candidate genes. Classification using 250 random genes was performed 100 times, and the mean AUC value was 0.5658. The *t*-test showed that the candidate genes significantly outperformed the randomly selected genes in classification with a *p* value of $5.723\times 10^{-52}$ (Fig. 4). As another comparison, we obtained 1,466 DEGs from the AD samples (GSE63060 and GSE63061 datasets) by the *t*-test and selected genes with a *q* value < 0.05 after Bonferroni correction. When these DEGs were used for AD classification in the ADNI cohort, the AUC value was 0.5757.

Furthermore, we examined serum glucose data in the ADNI dataset to consider the clinical characteristics of samples. When we compared glucose levels between the AD and the control groups by the *t*-test, there was no significant difference (*p* value = 0.513). In ADNI, there were 41 AD patients and 95 controls with high glucose levels, including prediabetes or diabetes samples ($\ge$ 100 mg/dL). Using the logistic regression model that were constructed for classification of AD (Table 7), we examined the classification performance for these hyperglycemic samples in the test set of each fold on tenfold cross-validation. The prediction performance for these hyperglycemic samples using the candidate genes (AUC = 0.715) was the highest among those using other genes (Supplementary Table S5). We also found that the predictive power of the prediction model using candidate genes was higher for these hyperglycemic samples compared to those for the whole samples (0.715 in Supplementary Table S5 and 0.6906 in Table 7, respectively).

We also performed classification using the independent T2D datasets (25 T2D and 71 controls). Among the 241 candidate genes, 179 genes were included in the T2D samples. On the threefold cross-validation, we obtained the AUC value of 0.9543. The AUC value of randomly selected 180 genes was 0.9458 and the predictive performances using other possible gene pairs were also high (Supplementary Table S6). This indicates that gene expression levels between T2D samples and controls in pancreatic islets were significantly different for most genes.

### Application of the proposed model to another AD dataset

We applied the proposed model to another AD dataset. For AD, ADNI was used instead of gene expression datasets of GSE63060 and GSE63061. For T2D, the same gene expression data (GSE78721) was used. When we try to find the optimal number of clusters for the ADNI dataset, the optimal rank k of the ADNI dataset was 2 (Supplementary Fig. S2). At least three clusters are required to determine significant differences in the distribution of each gene between clusters with the Kruskal-Wallis and Dunn test. Thus, as an alternative, we clustered the ADNI data with the non-optimal rank *k* = 3, resulting that 116 AD patients were clustered into three clusters with 17, 54, and 45 samples, respectively (Supplementary Table S7). When the pathway enrichment analysis was performed for genes in each cluster, the ADNI $M_3^+$ gene cluster contained the largest number of T2D-related pathways and followed by $M_1^+$. Among 41 hyperglycemic AD patients, the largest 19 belonged to ADNI S3. However, the proportion of hyperglycemic AD patients in each ADNI sample cluster was the highest in ADNI S1 followed by S3 (S1 = 0.47%, S2 = 0.259%, and S3 = 0.422%), which implies that the characteristics of patients of $S_1$ and $S_3$ are similar and can be merged for the high risk subgroup of T2D.

Additionally, there was no difference between the age of patients in $S_1$ and $S_3$, but the age of patients of $S_2$ was significantly lower than those of $S_1$ and $S_3$ (*p* values were 0.0013 and 0.0207 with a one-way ANOVA test, respectively). We also performed a one-way ANOVA test for APOE4 among subgroups of AD patients and observed no significant difference between APOE4 (*p* value as 0.35). Therefore, the proposed method may cluster patients that have some similar clinical characteristics such as the age, but not all of subgroups were clustered by these characteristics.

## Conclusion

We have provided a methodological and analytical approach for identifying correlations between AD and T2D at the genetic level. Since the AD dataset does not contain information about whether the AD patients have T2D or not, it is important to define subgroups of AD; the same is true for T2D. Because the conventional NMF is not suitable for this task, we developed a method of gene selection from gene expression data. After applying NMF to gene expression data, additional conditions were taken into account for detecting distinct characteristics of subgroups. Genes with significant differences in expression levels in each patient groups (AD and T2D) were first selected to screen patients with AD associated with T2D and patients with T2D associated with AD. We identified genes that characterize these specific AD and T2D patients and identified the potential relationship between the two diseases based on gene expression profiles. To validate these potential relationships from candidate genes, prediction errors of the classification between AD and controls from logistic regression were compared with randomly selected genes in an independent AD dataset. Inclusion of the candidate genes significantly increased the AUC values in classifying AD from controls compared with randomly selected genes.

In conclusion, we provide new insights for extracting differentially expressed genes with relative differences in a specific patient group. These genes were enriched with pathways related to both AD and T2D such as T cell selection and chemokine pathways. As AD patients have genetic heterogeneity, the investigation of commonly dysregulated pathways in AD and T2D can enhance personalized medical cares for a subgroup of AD. Further studies are needed to reveal the relationships among AD and other AD-related diseases which could improve the prevention and treatment of AD.

## Supplementary Information

Supplementary Information.
