# Supplementary material for: Correlation between Alzheimer’s disease and type 2 diabetes using non-negative matrix factorization
Source: Sci Rep. 2021 Jul 27;11:15265. doi: 10.1038/s41598-021-94048-0 (PMC8316581; doi:10.1038/s41598-021-94048-0)
Supplement: Supplementary file 1 — Supplementary Information. [file 41598_2021_94048_MOESM1_ESM.docx]

**Supplementary Materials for**

**Correlation between Alzheimer’s disease and type 2 diabetes using non-negative matrix factorization**

Yeonwoo Chung^1^ and Hyunju Lee^1,*^

^1^Gwangju Institute of Science and Technology, School of Electrical Engineering and Computer Science, Gwangju, Korea

*hyunjulee@gist.ac.kr

**Figure S1. Clustering results containing inappropriate gene.** The NMF completely decomposes the input expression matrix *A* so that the product of *W* and *H* is exactly same as the input matrix. The gene A,B and sample 1,2,3 are assigned to $G_{2}$, $G_{1}$, $S_{1}$, $S_{2}$, and $S_{3}$ using matrix *W* and *H*, respectively. In the input matrix *A*, the gene A which is assigned to $G_{2}$ using matrix *W* is relatively upregulated in $S_{2}$ compared to $S_{1}$ and $S_{3}$ in matrix *A*. However, gene B is assigned to $G_{1}$ even if there is no difference in expression level of gene B (0.38) between sample clusters.


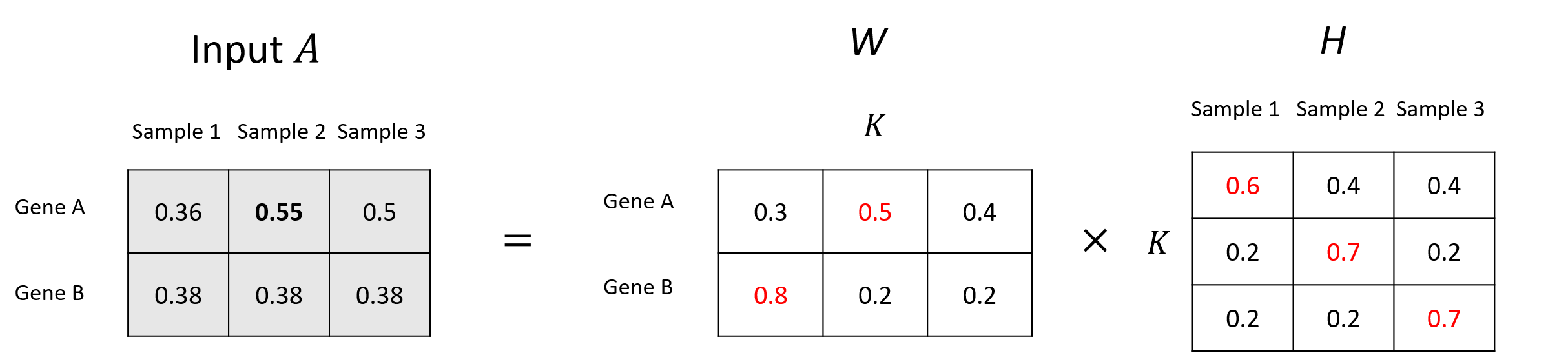


**Figure S2. Cophenetic correlation coefficients for the consensus matrix in the ADNI dataset**

**Table S1. Numbers of Alzheimer’s disease (AD) brain-related pathways in each AD differentially expressed gene module**

| AD $\boldsymbol{M}_{\boldsymbol{i}}$ | Enriched pathways | Common pathways with 160 AD brain-related pathways |
| --- | --- | --- |
| $\boldsymbol{M}_{\boldsymbol{1}}^{\boldsymbol{+}}$ | 16 | 1 |
| $\boldsymbol{M}_{\boldsymbol{1}}^{\boldsymbol{-}}$ | 10 | 1 |
| $\boldsymbol{M}_{\boldsymbol{2}}^{\boldsymbol{+}}$ | 30 | 1 |
| $\boldsymbol{M}_{\boldsymbol{2}}^{\boldsymbol{-}}$ | 101 | 28 |
| $\boldsymbol{M}_{\boldsymbol{3}}^{\boldsymbol{+}}$ | 205 | 6 |
| $\boldsymbol{M}_{\boldsymbol{3}}^{\boldsymbol{-}}$ | 164 | 54 |

**Table S2. Result of a one-way ANOVA for comparison of age on AD patient subgroups.**

|  | **Df** | **Sum Sq** | **Mean Sq** | **F value** | **Pr (>F)** |
| --- | --- | --- | --- | --- | --- |
| **Group** | 2 | 9 | 4.75 | 0.103 | 0.902 |
| **Residuals** | 282 | 13037 | 46.23 |  |  |

**R software (R version 3.6.1,** [**https://www.r-project.org/**](https://www.r-project.org/)**) was used for ANOVA.**

**Table S3. List of candidate genes**

| Gene symbol | Entrez ID | Gene name | Digsee AD | Digsee T2D | Alzgene | T2DiaCoD |
| --- | --- | --- | --- | --- | --- | --- |
| ABHD16A | 7920 | abhydrolase domain containing 16A, phospholipase | X | X | X | X |
| ACADVL | 37 | acyl-CoA dehydrogenase very long chain | X | X | X | X |
| ACTB | 60 | actin beta | X | X | X | X |
| ACTN1 | 87 | actinin alpha 1 | O | X | X | X |
| ACTR1A | 10121 | actin related protein 1A | X | X | X | X |
| ADAM9 | 8754 | ADAM metallopeptidase domain 9 | O | X | X | X |
| ADRM1 | 11047 | adhesion regulating molecule 1 | X | X | X | X |
| ADTRP | 84830 | androgen dependent TFPI regulating protein | X | X | X | X |
| AGMAT | 79814 | agmatinase | X | X | X | X |
| AHCYL1 | 10768 | adenosylhomocysteinase like 1 | X | X | X | X |
| AKIRIN2 | 55122 | akirin 2 | X | X | X | X |
| AKT1 | 207 | AKT serine/threonine kinase 1 | O | O | X | O |
| ALDOA | 226 | aldolase, fructose-bisphosphate A | X | X | X | X |
| ANKRD26 | 22852 | ankyrin repeat domain 26 | X | X | X | X |
| ANXA11 | 311 | annexin A11 | X | X | X | X |
| ANXA2R | 389289 | annexin A2 receptor | X | X | X | X |
| AP1M1 | 8907 | adaptor related protein complex 1 subunit mu 1 | X | X | X | X |
| APLP2 | 334 | amyloid beta precursor like protein 2 | O | X | X | X |
| APP | 351 | amyloid beta precursor protein | O | O | O | X |
| ARF1 | 375 | ADP ribosylation factor 1 | X | X | X | X |
| ARF3 | 377 | ADP ribosylation factor 3 | X | X | X | X |
| ARF5 | 381 | ADP ribosylation factor 5 | X | X | X | X |
| ARRB1 | 408 | arrestin beta 1 | X | X | X | X |
| ASAP1 | 50807 | ArfGAP with SH3 domain, ankyrin repeat and PH domain 1 | X | X | X | X |
| ATP6AP1 | 537 | ATPase H+ transporting accessory protein 1 | X | X | X | X |
| ATP6V0B | 533 | ATPase H+ transporting V0 subunit b | X | X | X | X |
| ATP6V0D1 | 9114 | ATPase H+ transporting V0 subunit d1 | X | X | X | X |
| ATP6V1B2 | 526 | ATPase H+ transporting V1 subunit B2 | X | X | X | X |
| BABAM2 | 9577 | BRISC and BRCA1 A complex member 2 | X | X | X | X |
| BCAS4 | 55653 | breast carcinoma amplified sequence 4 | X | X | X | X |
| BCL11A | 53335 | BAF chromatin remodeling complex subunit BCL11A | X | O | X | X |
| BICD2 | 23299 | BICD cargo adaptor 2 | X | X | X | X |
| BTLA | 151888 | B and T lymphocyte associated | X | X | X | X |
| C1RL | 51279 | complement C1r subcomponent like | X | X | X | X |
| CALCOCO2 | 10241 | calcium binding and coiled-coil domain 2 | O | X | X | X |
| CAP1 | 10487 | cyclase associated actin cytoskeleton regulatory protein 1 | X | X | X | X |
| CAPZB | 832 | capping actin protein of muscle Z-line subunit beta | X | X | X | X |
| CARS1 | 833 | cysteinyl-tRNA synthetase 1 | X | X | X | X |
| CBLL1 | 79872 | Cbl proto-oncogene like 1 | X | X | X | X |
| CCNI | 10983 | cyclin I | X | X | X | X |
| CCNK | 8812 | cyclin K | X | X | X | X |
| CCNY | 219771 | cyclin Y | X | X | X | X |
| CD14 | 929 | CD14 molecule | O | O | O | X |
| CD160 | 11126 | CD160 molecule | X | X | X | X |
| CD302 | 9936 | CD302 molecule | X | X | X | X |
| CD3E | 916 | CD3e molecule | X | X | X | X |
| CD3G | 917 | CD3g molecule | X | X | X | X |
| CD6 | 923 | CD6 molecule | X | X | X | X |
| CD79B | 974 | CD79b molecule | X | X | X | X |
| CEBPB | 1051 | CCAAT enhancer binding protein beta | O | X | O | X |
| CELA1 | 1990 | chymotrypsin like elastase 1 | X | X | X | X |
| CELF2 | 10659 | CUGBP Elav-like family member 2 | O | X | O | X |
| CERS2 | 29956 | ceramide synthase 2 | X | X | X | X |
| CFLAR | 8837 | CASP8 and FADD like apoptosis regulator | O | X | X | X |
| CHMP1B | 57132 | charged multivesicular body protein 1B | X | X | X | X |
| CLP1 | 10978 | cleavage factor polyribonucleotide kinase subunit 1 | X | X | X | X |
| CNPY3 | 10695 | canopy FGF signaling regulator 3 | X | X | X | X |
| COPG1 | 22820 | COPI coat complex subunit gamma 1 | X | X | X | X |
| CPQ | 10404 | carboxypeptidase Q | X | X | X | X |
| CREBBP | 1387 | CREB binding protein | O | O | X | X |
| CRK | 1398 | CRK proto-oncogene, adaptor protein | X | X | X | X |
| CSNK1D | 1453 | casein kinase 1 delta | O | X | X | X |
| CTDNEP1 | 23399 | CTD nuclear envelope phosphatase 1 | X | X | X | X |
| CTDSP2 | 10106 | CTD small phosphatase 2 | X | X | X | X |
| CTLA4 | 1493 | cytotoxic T-lymphocyte associated protein 4 | X | O | X | X |
| CTNNA1 | 1495 | catenin alpha 1 | X | X | X | X |
| CUX1 | 1523 | cut like homeobox 1 | X | X | X | X |
| DBNL | 28988 | drebrin like | O | X | X | X |
| DCP1A | 55802 | decapping mRNA 1A | X | X | X | X |
| DEF8 | 54849 | differentially expressed in FDCP 8 homolog | X | X | X | X |
| DEGS1 | 8560 | delta 4-desaturase, sphingolipid 1 | X | X | X | X |
| DENND5A | 23258 | DENN domain containing 5A | X | X | X | X |
| DHRS7 | 51635 | dehydrogenase/reductase 7 | X | X | X | X |
| DNAJB12 | 54788 | DnaJ heat shock protein family (Hsp40) member B12 | X | X | X | X |
| DNAJB6 | 10049 | DnaJ heat shock protein family (Hsp40) member B6 | X | X | X | X |
| DNTTIP1 | 116092 | deoxynucleotidyltransferase terminal interacting protein 1 | X | X | X | X |
| DPH6 | 89978 | diphthamine biosynthesis 6 | X | X | X | X |
| EIF4G3 | 8672 | eukaryotic translation initiation factor 4 gamma 3 | X | X | X | X |
| ELOVL4 | 6785 | ELOVL fatty acid elongase 4 | X | X | X | X |
| EVI5 | 7813 | ecotropic viral integration site 5 | X | X | X | X |
| F11R | 50848 | F11 receptor | X | X | O | X |
| FKBP15 | 23307 | FKBP prolyl isomerase family member 15 | X | X | X | X |
| FLII | 2314 | FLII actin remodeling protein | X | X | X | X |
| FLOT1 | 10211 | Flotillin 1 | O | X | O | X |
| GLYR1 | 84656 | glyoxylate reductase 1 homolog | X | X | X | X |
| GNB2 | 2783 | G protein subunit beta 2 | X | X | X | X |
| GNG5 | 2787 | G protein subunit gamma 5 | X | X | X | X |
| GNS | 2799 | glucosamine (N-acetyl)-6-sulfatase | X | X | X | X |
| GPR108 | 56927 | G protein-coupled receptor 108 | X | X | X | X |
| GRAP | 10750 | GRB2 related adaptor protein | X | X | X | X |
| GTF3C3 | 9330 | general transcription factor IIIC subunit 3 | X | X | X | X |
| GYG1 | 2992 | glycogenin 1 | X | X | X | X |
| HADHB | 3032 | hydroxyacyl-CoA dehydrogenase trifunctional multienzyme complex subunit beta | X | X | X | X |
| HLA-E | 3133 | major histocompatibility complex, class I, E | O | X | X | X |
| HNRNPK | 3190 | heterogeneous nuclear ribonucleoprotein K | X | X | X | X |
| IDS | 3423 | iduronate 2-sulfatase | X | X | X | X |
| IFITM2 | 10581 | interferon induced transmembrane protein 2 | X | X | X | X |
| IFNGR2 | 3460 | interferon gamma receptor 2 | X | X | X | X |
| IL10RB | 3588 | interleukin 10 receptor subunit beta | X | X | X | X |
| IL13RA1 | 3597 | interleukin 13 receptor subunit alpha 1 | X | X | X | X |
| IL23A | 51561 | interleukin 23 subunit alpha | X | X | X | X |
| ILK | 3611 | integrin linked kinase | X | O | X | O |
| IRF2 | 3660 | interferon regulatory factor 2 | O | X | X | X |
| IRF2BPL | 64207 | interferon regulatory factor 2 binding protein like | X | X | X | X |
| IST1 | 9798 | IST1 factor associated with ESCRT-III | X | X | X | X |
| JAK1 | 3716 | Janus kinase 1 | X | O | X | X |
| KAT8 | 84148 | lysine acetyltransferase 8 | X | X | X | X |
| KBTBD3 | 143879 | kelch repeat and BTB domain containing 3 | X | X | X | X |
| KIAA2013 | 90231 | KIAA2013 | X | X | X | X |
| KLF12 | 11278 | Kruppel like factor 12 | X | X | X | X |
| KLHL3 | 26249 | kelch like family member 3 | X | X | X | X |
| KPNA5 | 3841 | karyopherin subunit alpha 5 | X | X | X | X |
| KPNB1 | 3837 | karyopherin subunit beta 1 | X | X | X | X |
| LAGE3 | 8270 | L antigen family member 3 | X | X | X | X |
| LAMP1 | 3916 | lysosomal associated membrane protein 1 | O | X | X | X |
| LAX1 | 54900 | lymphocyte transmembrane adaptor 1 | X | X | X | X |
| LEF1 | 51176 | lymphoid enhancer binding factor 1 | O | X | X | X |
| LRRFIP2 | 9209 | LRR binding FLII interacting protein 2 | X | X | X | X |
| LTBR | 4055 | lymphotoxin beta receptor | X | X | X | X |
| LY9 | 4063 | lymphocyte antigen 9 | X | X | X | X |
| MAL | 4118 | mal, T cell differentiation protein | X | X | X | X |
| MAP4K4 | 9448 | mitogen-activated protein kinase kinase kinase kinase 4 | X | X | X | X |
| MARCKS | 4082 | myristoylated alanine rich protein kinase C substrate | O | X | X | X |
| METRNL | 284207 | meteorin like, glial cell differentiation regulator | X | X | X | X |
| MFSD14A | 64645 | major facilitator superfamily domain containing 14A | X | X | X | X |
| MFSD14B | 84641 | major facilitator superfamily domain containing 14B | X | X | X | X |
| MGAT1 | 4245 | alpha-1,3-mannosyl-glycoprotein 2-beta-N-acetylglucosaminyltransferase | X | X | X | X |
| MIDEAS | 91748 | mitotic deacetylase associated SANT domain protein | X | X | X | X |
| MOB3A | 126308 | MOB kinase activator 3A | X | X | X | X |
| MSN | 4478 | moesin | X | O | X | X |
| MVP | 9961 | major vault protein | X | X | X | X |
| MYB | 4602 | MYB proto-oncogene, transcription factor | X | O | X | X |
| MYH9 | 4627 | myosin heavy chain 9 | X | O | X | X |
| NDEL1 | 81565 | nudE neurodevelopment protein 1 like 1 | X | X | X | X |
| NEMP1 | 23306 | nuclear envelope integral membrane protein 1 | X | X | X | X |
| OS9 | 10956 | OS9 endoplasmic reticulum lectin | X | X | X | X |
| PAF1 | 54623 | PAF1 homolog, Paf1/RNA polymerase II complex component | X | X | X | X |
| PCBP1 | 5093 | poly(rC) binding protein 1 | X | X | X | X |
| PELO | 53918 | pelota mRNA surveillance and ribosome rescue factor | X | X | X | X |
| PGAM1 | 5223 | phosphoglycerate mutase 1 | X | X | O | X |
| PGD | 5226 | phosphogluconate dehydrogenase | O | X | X | X |
| PGK1 | 5230 | phosphoglycerate kinase 1 | O | X | X | X |
| PHC2 | 1912 | polyhomeotic homolog 2 | X | X | X | X |
| PHF23 | 79142 | PHD finger protein 23 | X | X | X | X |
| PI4KB | 5298 | phosphatidylinositol 4-kinase beta | X | X | X | X |
| PICALM | 8301 | phosphatidylinositol binding clathrin assembly protein | O | X | X | X |
| PIM3 | 415116 | Pim-3 proto-oncogene, serine/threonine kinase | X | X | X | X |
| PITPNA | 5306 | phosphatidylinositol transfer protein alpha | X | X | X | X |
| PLAG1 | 5324 | PLAG1 zinc finger | X | X | X | X |
| PLEKHB2 | 55041 | pleckstrin homology domain containing B2 | X | X | X | X |
| PLOD1 | 5351 | procollagen-lysine,2-oxoglutarate 5-dioxygenase 1 | O | X | X | X |
| PPT1 | 5538 | palmitoyl-protein thioesterase 1 | X | X | X | X |
| PRCC | 5546 | proline rich mitotic checkpoint control factor | X | X | X | X |
| PRCP | 5547 | prolylcarboxypeptidase | X | O | X | X |
| PRKCD | 5580 | protein kinase C delta | O | O | X | X |
| PRMT5 | 10419 | protein arginine methyltransferase 5 | X | X | X | X |
| PSAP | 5660 | prosaposin | X | O | O | X |
| PTTG1IP | 754 | PTTG1 interacting protein | X | X | X | X |
| PYHIN1 | 149628 | pyrin and HIN domain family member 1 | X | X | X | X |
| RAB5C | 5878 | RAB5C, member RAS oncogene family | X | X | X | X |
| RAB5IF | 55969 | RAB5 interacting factor | X | X | X | X |
| RAB7A | 7879 | RAB7A, member RAS oncogene family | O | O | X | X |
| RAC1 | 5879 | Rac family small GTPase 1 | O | O | X | X |
| RADX | 55086 | RPA1 related single stranded DNA binding protein, X-linked | X | X | X | X |
| RAF1 | 5894 | Raf-1 proto-oncogene, serine/threonine kinase | O | O | X | X |
| RALB | 5899 | RAS like proto-oncogene B | O | X | X | X |
| RALBP1 | 10928 | ralA binding protein 1 | X | X | X | X |
| RALGAPA1 | 253959 | Ral GTPase activating protein catalytic subunit alpha 1 | X | X | X | X |
| RALY | 22913 | RALY heterogeneous nuclear ribonucleoprotein | X | X | X | X |
| RASGRP1 | 10125 | RAS guanyl releasing protein 1 | X | X | X | X |
| RBM22 | 55696 | RNA binding motif protein 22 | X | X | X | X |
| RBMS1 | 5937 | RNA binding motif single stranded interacting protein 1 | X | O | X | X |
| RELA | 5970 | RELA proto-oncogene, NF-kB subunit | X | O | X | X |
| RGL2 | 5863 | ral guanine nucleotide dissociation stimulator like 2 | X | X | X | X |
| RHBDD2 | 57414 | rhomboid domain containing 2 | X | X | X | X |
| RHOA | 387 | ras homolog family member A | O | O | X | O |
| RIC8A | 60626 | RIC8 guanine nucleotide exchange factor A | X | X | X | X |
| RILPL2 | 196383 | Rab interacting lysosomal protein like 2 | X | X | X | X |
| RNF130 | 55819 | ring finger protein 130 | X | X | X | X |
| RNF135 | 84282 | ring finger protein 135 | X | X | X | X |
| RNF4 | 6047 | ring finger protein 4 | X | X | X | X |
| RNPEP | 6051 | arginyl aminopeptidase | X | X | X | X |
| RRAGC | 64121 | Ras related GTP binding C | X | X | X | X |
| RRBP1 | 6238 | ribosome binding protein 1 | X | X | X | X |
| RTF2 | 51507 | replication termination factor 2 | X | X | X | X |
| RTN3 | 10313 | reticulon 3 | O | X | X | X |
| S100A11 | 6282 | S100 calcium binding protein A11 | O | X | X | X |
| SBF2 | 81846 | SET binding factor 2 | X | X | X | X |
| SDCBP | 6386 | syndecan binding protein | X | X | X | X |
| SERINC3 | 10955 | serine incorporator 3 | X | X | X | X |
| SH3GLB1 | 51100 | SH3 domain containing GRB2 like, endophilin B1 | X | X | X | X |
| SIRPG | 55423 | signal regulatory protein gamma | X | O | X | X |
| SLAMF6 | 114836 | SLAM family member 6 | X | X | X | X |
| SLC25A42 | 284439 | solute carrier family 25 member 42 | X | X | X | X |
| SNAP23 | 8773 | synaptosome associated protein 23 | X | X | X | X |
| SQOR | 58472 | sulfide quinone oxidoreductase | X | X | X | X |
| SQSTM1 | 8878 | sequestosome 1 | O | O | X | X |
| STAT3 | 6774 | signal transducer and activator of transcription 3 | O | O | X | X |
| STAT4 | 6775 | signal transducer and activator of transcription 4 | X | O | X | X |
| STAT6 | 6778 | signal transducer and activator of transcription 6 | X | O | X | X |
| STAU1 | 6780 | staufen double-stranded RNA binding protein 1 | X | X | X | X |
| TAF4 | 6874 | TATA-box binding protein associated factor 4 | X | X | X | X |
| TAGLN2 | 8407 | transgelin 2 | X | X | X | X |
| TAX1BP3 | 30851 | Tax1 binding protein 3 | X | X | X | X |
| TGFBR2 | 7048 | transforming growth factor beta receptor 2 | O | O | X | X |
| THEMIS | 387357 | thymocyte selection associated | X | X | X | X |
| TIMP2 | 7077 | TIMP metallopeptidase inhibitor 2 | O | O | X | O |
| TLN1 | 7094 | talin 1 | X | X | X | X |
| TMBIM1 | 64114 | transmembrane BAX inhibitor motif containing 1 | X | X | X | X |
| TMBIM6 | 7009 | transmembrane BAX inhibitor motif containing 6 | X | X | X | X |
| TMCO3 | 55002 | transmembrane and coiled-coil domains 3 | X | X | X | X |
| TMEM156 | 80008 | transmembrane protein 156 | X | X | X | X |
| TMEM184B | 25829 | transmembrane protein 184B | X | X | X | X |
| TMEM43 | 79188 | transmembrane protein 43 | X | X | X | X |
| TMX4 | 56255 | thioredoxin related transmembrane protein 4 | X | X | X | X |
| TNFRSF10B | 8795 | TNF receptor superfamily member 10b | O | X | X | X |
| TNFRSF1A | 7132 | TNF receptor superfamily member 1A | O | O | O | X |
| TPD52L2 | 7165 | TPD52 like 2 | X | X | X | X |
| TPP1 | 1200 | tripeptidyl peptidase 1 | X | X | X | X |
| TSC22D3 | 1831 | TSC22 domain family member 3 | X | X | X | X |
| TSEN34 | 79042 | tRNA splicing endonuclease subunit 34 | X | X | X | X |
| UBAP1 | 51271 | ubiquitin associated protein 1 | X | X | X | X |
| UBE2G1 | 7326 | ubiquitin conjugating enzyme E2 G1 | X | X | X | X |
| UBE2M | 9040 | ubiquitin conjugating enzyme E2 M | O | X | X | X |
| UBR4 | 23352 | ubiquitin protein ligase E3 component n-recognin 4 | X | X | X | X |
| UQCC2 | 84300 | ubiquinol-cytochrome c reductase complex assembly factor 2 | X | X | X | X |
| USP48 | 84196 | ubiquitin specific peptidase 48 | X | X | X | X |
| VCP | 7415 | valosin containing protein | O | X | O | X |
| WDR1 | 9948 | WD repeat domain 1 | X | X | X | X |
| WDR13 | 64743 | WD repeat domain 13 | X | X | X | X |
| WDR92 | 116143 | WD repeat domain 92 | X | X | X | X |
| WIPI2 | 26100 | WD repeat domain, phosphoinositide interacting 2 | X | X | X | X |
| YWHAB | 7529 | tyrosine 3-monooxygenase/tryptophan 5-monooxygenase activation protein beta | X | X | X | X |
| ZDHHC5 | 25921 | zinc finger DHHC-type palmitoyltransferase 5 | X | X | X | X |
| ZDHHC7 | 55625 | zinc finger DHHC-type palmitoyltransferase 7 | X | X | X | X |
| ZFP36L2 | 678 | ZFP36 ring finger protein like 2 | X | X | X | X |
| ZMIZ1 | 57178 | zinc finger MIZ-type containing 1 | X | O | X | X |
| ZNF106 | 64397 | zinc finger protein 106 | X | X | X | X |
| ZNF365 | 22891 | zinc finger protein 365 | X | X | X | X |
| ZNF616 | 90317 | zinc finger protein 616 | X | X | X | X |
| ZNF841 | 284371 | zinc finger protein 841 | X | X | X | X |

**Table S4. Functional enrichment analysis of the candidate genes**

| Genes in function | Functional genes in candidate genes | Description | P-value | Adjusted p-value(<0.05) |
| --- | --- | --- | --- | --- |
| 255 | 18 | GO_POSITIVE_REGULATION_OF_CELL_CELL_ADHESION | 3.79E-09 | 2.85E-05 |
| 70 | 9 | KEGG_PANCREATIC_CANCER | 2.17E-07 | 4.03E-05 |
| 47 | 9 | GO_T_CELL_SELECTION | 5.83E-09 | 4.39E-05 |
| 34 | 8 | GO_POSITIVE_T_CELL_SELECTION | 7.33E-09 | 5.52E-05 |
| 215 | 16 | GO_POSITIVE_REGULATION_OF_LEUKOCYTE_CELL_CELL_ADHESION | 1.35E-08 | 0.000101928 |
| 238 | 16 | GO_T_CELL_DIFFERENTIATION | 5.64E-08 | 0.000425056 |
| 121 | 10 | KEGG_LYSOSOME | 2.93E-06 | 0.000544795 |
| 154 | 12 | GO_AZUROPHIL_GRANULE | 5.59E-07 | 0.000558308 |
| 73 | 8 | KEGG_ADHERENS_JUNCTION | 3.57E-06 | 0.000663158 |
| 105 | 10 | GO_PIGMENT_GRANULE | 7.95E-07 | 0.000794374 |
| 136 | 12 | GO_ALPHA_BETA_T_CELL_ACTIVATION | 1.44E-07 | 0.001087221 |
| 68 | 7 | KEGG_EPITHELIAL_CELL_SIGNALING_IN_HELICOBACTER_PYLORI_INFECTION | 2.23E-05 | 0.004142846 |
| 188 | 11 | KEGG_CHEMOKINE_SIGNALING_PATHWAY | 2.58E-05 | 0.004806622 |
| 155 | 10 | KEGG_JAK_STAT_SIGNALING_PATHWAY | 2.62E-05 | 0.004876146 |
| 26 | 6 | GO_T_CELL_LINEAGE_COMMITMENT | 6.73E-07 | 0.005065672 |
| 54 | 6 | KEGG_VIBRIO_CHOLERAE_INFECTION | 5.63E-05 | 0.010475938 |
| 172 | 11 | GO_VACUOLAR_LUMEN | 1.12E-05 | 0.011236912 |
| 108 | 8 | KEGG_T_CELL_RECEPTOR_SIGNALING_PATHWAY | 6.41E-05 | 0.011931807 |
| 17 | 5 | GO_CD4_POSITIVE_ALPHA_BETA_T_CELL_LINEAGE_COMMITMENT | 1.61E-06 | 0.012119437 |
| 91 | 9 | GO_CD4_POSITIVE_ALPHA_BETA_T_CELL_ACTIVATION | 2.08E-06 | 0.015652077 |
| 295 | 14 | GO_SECRETORY_GRANULE_MEMBRANE | 2.23E-05 | 0.022313115 |
| 62 | 6 | KEGG_COLORECTAL_CANCER | 0.000123344 | 0.022942068 |
| 124 | 9 | GO_FICOLIN_1_RICH_GRANULE | 2.63E-05 | 0.026302242 |
| 20 | 5 | GO_CD4_POSITIVE_OR_CD8_POSITIVE_ALPHA_BETA_T_CELL_LINEAGE_COMMITMENT | 3.91E-06 | 0.029447379 |
| 100 | 9 | GO_ALPHA_BETA_T_CELL_DIFFERENTIATION | 4.58E-06 | 0.034458571 |
| 126 | 8 | KEGG_NEUROTROPHIN_SIGNALING_PATHWAY | 0.000188801 | 0.035116995 |
| 96 | 7 | KEGG_FC_GAMMA_R_MEDIATED_PHAGOCYTOSIS | 0.00020364 | 0.037877102 |
| 132 | 8 | KEGG_TIGHT_JUNCTION | 0.000259645 | 0.048293944 |
| 17 | 4 | GO_PSEUDOPODIUM | 5.00E-05 | 0.04998667 |

**Table S5. Classification performance of Alzheimer's disease (AD) and controls for hyperglycemic samples from the ADNI cohort using different sets of genes. (***M_i_***,** *M_j_***) represents the common genes between the AD** *M_i_* **module and type 2 diabetes (T2D)** *M_j_* **module used for classification.**

| **( AD** $\boldsymbol{M}_{\boldsymbol{i}}$**, T2D** $\boldsymbol{M}_{\boldsymbol{j}}$ **)** | **AUC** |
| --- | --- |
| $(M_{1}$,$M_{1}$) | 0.5055 |
| $(M_{1}$,$M_{2}$) | 0.5584 |
| $(M_{1}$,$M_{3}$) | 0.5373 |
| $(M_{2}$,$M_{1}$) | 0.5227 |
| $(M_{2}$,$M_{2}$) | 0.5214 |
| $(M_{2}$,$M_{3}$) | 0.5899 |
| $(M_{3}$,$M_{1}$) | 0.5332 |
| $(M_{3}$,$M_{2}$) | 0.5576 |
| $(M_{3}$,$M_{3}$) | 0.7150 |

**Table S6. Performance of classification of type 2 diabetes (T2D) and controls in the independent merged T2D dataset using different sets of genes. (**$\boldsymbol{M}_{\boldsymbol{i}}\boldsymbol{,}\boldsymbol{M}_{\boldsymbol{j}}$**) represents the common genes between the Alzheimer’s disease (AD)** $\boldsymbol{M}_{\boldsymbol{i}}$ **module and T2D** $\boldsymbol{M}_{\boldsymbol{j}}$ **module used for classification**

| **( AD** $\boldsymbol{M}_{\boldsymbol{i}}$**, T2D** $\boldsymbol{M}_{\boldsymbol{j}}$ **)** | **AUC** |
| --- | --- |
| $(M_{1}$,$M_{1}$) | 0.9152 |
| $(M_{1}$,$M_{2}$) | $\mathrm{NA}^{*}$ |
| $(M_{1}$,$M_{3}$) | 0.9597 |
| $(M_{2}$,$M_{1}$) | 0.7472 |
| $(M_{2}$,$M_{2}$) | 0.9225 |
| $(M_{2}$,$M_{3}$) | 0.8772 |
| $(M_{3}$,$M_{1}$) | 0.8312 |
| $(M_{3}$,$M_{2}$) | 0.8841 |
| $(M_{3}$,$M_{3}$) | 0.9543 |

$\mathrm{NA}^{*}$: Expression values of genes were not present in the merged T2D dataset.

**Table S7. Numbers of DEGs in each cluster when clustering the ADNI dataset**

| **Cluster *i*** | $\boldsymbol{S}_{\boldsymbol{i}}$ | $\boldsymbol{M}_{\boldsymbol{i}}^{\boldsymbol{+}}$ | $\boldsymbol{M}_{\boldsymbol{i}}^{\boldsymbol{-}}$ |
| --- | --- | --- | --- |
| 1 | 17 | 65 | 45 |
| 2 | 54 | 1221 | 928 |
| 3 | 45 | 1182 | 1415 |
